# Does a Red House Affect Rhythms in Mice with a Corrupted Circadian System?

**DOI:** 10.3390/ijms22052288

**Published:** 2021-02-25

**Authors:** Menekse Öztürk, Marc Ingenwerth, Martin Sager, Charlotte von Gall, Amira A. H. Ali

**Affiliations:** 1 Institute for Anatomy II, Medical Faculty, Heinrich-Heine-University, Moorenstrasse 5, 40225 Dusseldorf, Germany; menekse.oeztuerk@hhu.de (M.Ö.); Marc.Ingenwerth@uk-essen.de (M.I.); amira.ali@med.uni-duesseldorf.de (A.A.H.A.); 2 Institute of Pathology, Medical Faculty, University Duisburg-Essen, Hufelandstrasse 55, 45147 Essen, Germany; 3 Central Institute for Animal Research and Animal Protection (ZETT), Medical Faculty, Heinrich Heine University, Moorenstrasse 5, 40225 Dusseldorf, Germany; Martin.Sager@med.uni-duesseldorf.de

**Keywords:** circadian, mammalian, suprachiasmatic nucleus, entrainment, synchronization, red house, Bmal1, masking

## Abstract

The circadian rhythms of body functions in mammals are controlled by the circadian system. The suprachiasmatic nucleus (SCN) in the hypothalamus orchestrates subordinate oscillators. Time information is conveyed from the retina to the SCN to coordinate an organism’s physiology and behavior with the light/dark cycle. At the cellular level, molecular clockwork composed of interlocked transcriptional/translational feedback loops of clock genes drives rhythmic gene expression. Mice with targeted deletion of the essential clock gene *Bmal1* (Bmal1−/−) have an impaired light input pathway into the circadian system and show a loss of circadian rhythms. The red house (RH) is an animal welfare measure widely used for rodents as a hiding place. Red plastic provides light at a low irradiance and long wavelength—conditions which affect the circadian system. It is not known yet whether the RH affects rhythmic behavior in mice with a corrupted circadian system. Here, we analyzed whether the RH affects spontaneous locomotor activity in Bmal1−/− mice under standard laboratory light conditions. In addition, mPER1- and p-ERK-immunoreactions, as markers for rhythmic SCN neuronal activity, and day/night plasma corticosterone levels were evaluated. Our findings indicate that application of the RH to Bmal1−/− abolishes rhythmic locomotor behavior and dampens rhythmic SCN neuronal activity. However, RH had no effect on the day/night difference in corticosterone levels.

## 1. Introduction

In mammals, many body rhythms, such as the sleep-wake cycle or hormone secretions, oscillate within a period of approximately 24 h under constant environmental conditions—this is called a circadian rhythm. These circadian rhythms are orchestrated by a circadian rhythm generator, which is located in the suprachiasmatic nucleus (SCN) of the hypothalamus [1]. The SCN receives information about the environmental time via the retina and controls rhythmic bodily functions via the autonomous nervous system and the endocrine system. Glucocorticoids such as corticosterone show a circadian fluctuation peaking at the beginning of the activity phase [2,3] and play an important role in coordinating circadian oscillators within the body. In addition, plasma corticosterone is a reliable indicator of stress in mice [4]; therefore, it is used in animal welfare evaluations [5].

The intracellular clock mechanism that drives rhythmic gene expression, and thus rhythmic cell and organ function, is based on interacting positive and negative transcriptional-translational feedback loops of clock genes [6]. Two basic helix-loop-helix (bHLH)/PAS-containing transcription factors, CLOCK and BMAL1, provide the basic drive to the system by activating transcription through E box enhancers [7,8]. CLOCK:BMAL1 heterodimers drive the transcription of three period genes (mPer1–3) and two cryptochrome genes (mCry1–2) [7,9,10]. The mPER and mCRY proteins form complexes, which translocate to the nucleus and interact with CLOCK:BMAL1 heterodimers to inhibit transcription, thus closing the feedback loop [11]. This clockwork is not only present in SCN cells, but also in the retina and other brain regions, as well as peripheral tissues. The retina clock [12,13] controls various aspects of retinal development and photoreceptor viability [14,15], as well as retinal physiology [16].

Light is the most effective zeitgeber [17]; it entrains the period length and the phase of circadian rhythms to the environmental light/dark (LD) cycle [6]. Light/dark information received by the eye is conveyed to the SCN by glutamate and pituitary adenylate cyclase-activating peptide (PACAP) [18,19]. Activation of the respective receptors induces signal transduction pathways, resulting in the phosphorylation of the transcription factor CREB [20,21,22] and the kinases ERK1/2 [23], and then the subsequent light-induced CRE-dependent activation of *Per* expression leads to adjustment of the SCN molecular clockwork [24]. Moreover, masking of light and darkness can override or alter the influence of the circadian timekeeping system on behavior and physiology and play an important role in shaping the daily pattern of activity. Nocturnal animals generally become more active in response to darkness (positive masking) and less active in response to light (negative masking) [25,26]. It is still a matter of debate whether masking is modulated by SCN output to downstream hypothalamic nuclei, or by a direct effect of light on activity [27]. Circadian photoentrainment behavior of den dwelling nocturnal rodents is illustrated most impressively by experiments using a simulated den [28]. In a simulated den, the animals have access to a dark box containing nesting material and an activity box with a running wheel which can be illuminated; both compartments are connected by a porthole. In this setting, the animals spend their entire rest period (approximately 12 h) in the dark box. Upon arousal, they leave the dark box to actively sample light information from the porthole. In darkness, they enter the activity box and initiate wheel running. Light at arousal time results in a retreat to the dark box and a phase delay in activity onset on subsequent days. This experiment emphasized the desire of the animals to expose themselves to light as little as possible, and as much as needed.

Three retinal photoreceptor classes—the rods, the cones, and the intrinsically photosensitive retinal ganglion cells (ipRGCs)—contribute light information to the circadian system [29,30,31]. The ipRGCs encode ambient light (irradiance) for the circadian system and are activated both by their intrinsic melanopsin-dependent phototransduction cascade and by the rods and cones [32]. The mouse retina contains different visual pigments: the rod opsins with a peak sensitivity at 498 nm, the L- and the S-cone opsins with a peak in sensitivity at 505 and ~360 nm, respectively, and the iRGCs pigment melanopsin, with a peak in sensitivity at 484 nm [33]. While melanopsin appears to be the primary photopigment produced in response to long-duration light exposure and at higher irradiances, cone photoreceptors contribute substantially to nonvisual responses at the beginning of light exposure and at lower irradiances [34]. Consistently, longer-wavelength light has a stronger effect on the mammalian circadian system than short-wavelength light, as light with a higher wavelength (yellow) lengthens the circadian period reliably and is more effective at re-entraining the circadian system as compared to light with a shorter wavelength (blue) [35]. This is expedient, as during twilight, when the circadian system is especially sensitive to changes in the length of the light and the dark phase during the seasons, the intensity of the illumination decreases and the spectral composition shifts towards longer wavelengths [36].

In mice with a targeted deletion of the essential clock gene Bmal1 (Bmal1−/−), the molecular clockwork is disrupted and the mice are arrhythmic in constant darkness [37]. Bmal1−/− mice show various aspects of premature aging and a reduced lifespan (around 9 months) [38]. Importantly, in Bmal1−/− mice, the light input into the circadian system is compromised [14,39,40]. Electroretinography in mice with a retina-specific deletion of Bmal1 show a reduction in b-wave amplitudes under both light- and dark-adapted conditions [14,41], indicating that visual information processing in both the rod and cone pathways is affected [14]. This also resembles the phenotype of mice lacking melanopsin [41], the photopigment of intrinsically photosensitive retinal ganglion cells. Moreover, removal of Bmal1 from the retina affects morphology, accelerates the decline of visual functions during aging, and reduces the viability of cone photoreceptors [14]. Thus, aged Bmal1-deficient mice are a well-established model for severely affected retinal function.

Constant efforts are being made to improve animal welfare and housing conditions of laboratory animals [42,43,44,45], including their growth, physiological parameters (e.g., hormones), and behavioral activity [46]. Accordingly, application of nesting materials or objects that provide a secure location and a dark place, such as the red house (RH) [46,47], have been widely introduced. However, the RH is not comparable to a den, as it does not allow the animals to move within it. More importantly, the RH is not a dark box, but is illuminated with light at a low irradiance and long wavelength—conditions resembling twilight light conditions, which are now known to affect the circadian system. Thus, validation is required to avoid interference with experimental outcomes. Therefore, we tested whether the application of the RH affects rhythmic locomotor activity and rhythmic SCN neuronal activity and day/night changes in corticosterone levels in wildtype and Bmal1−/− mice, which are more prone to perturbations of the light regime.

## 2. Results

### 2.1. Spontaneous Locomotor Activity Is Affected by RH

**Figure 1:**
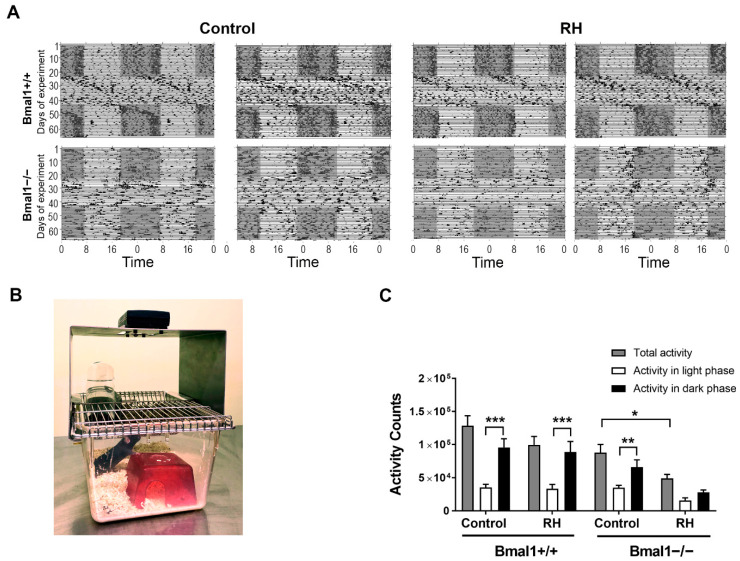
(**A**) Representative double plotted actograms of spontaneous locomotor activity of Bmal1+/+ and Bmal1−/− mice kept without (control, left panel) or with a red house (RH, right panel) under a 12-h light/12-h dark cycle (LD), followed by a constant light cycle (LL) and a second LD. Black bars represent activity. Grey boxes indicate periods of darkness. (**B**) Representative picture showing the red house and on-cage activity monitoring. (**C**) Mean total activity counts (grey bars), during the light phase in control (white bars) and the corresponding dark phases (black bars), under the second LD. Values are shown as mean + SEM.* *p* < 0.05, ** *p* < 0.01, *** *p* < 0.001. Groups were compared using the Mann-Whitney U-test (Bmal1+/+: *n* = 12 control, *n* = 11 with RH; Bmal1−/−: *n* = 8 per group).

Under constant light (LL), circadian spontaneous locomotor activity was disrupted in both genotypes. In Bmal1+/+ mice, the power of the 24-h phase gradually decreases during the first 10 days in LL (Figure S1B). In Bmal1−/− mice, the power of the 24-h phase was significantly lower in the RH group than the control group (Figure S1C). This shows that the RH enhances the effect of constant light on rhythm instability in Bmal1-deficient mice. In Bmal1+/+ mice, there was no difference in total activity between control mice (*n* = 12: 6 female and 6 male) and mice with the RH (*n* = 11: 6 female and 5 male). However, in Bmal1−/− mice, total locomotor activity was reduced in mice with the RH as compared to controls (*p* = 0.004) (Figure S1). Under LD following LL, Bmal1+/+ mice showed higher spontaneous locomotor activity during the dark phase than during the light phase in both the control (*p* = 0.0001) and the RH group (*p* = 0.0001). Additionally, the Bmal1−/− control mice (*n* = 9: 4 female and 5 male) showed higher spontaneous locomotor activity during the dark phase than during the light phase (*p* = 0.03). In contrast, Bmal1−/− mice with an RH (*n* = 8: 3 female and 5 male) showed a tendency to be more active during the dark phase than during the light phase, but this difference was not statistically significant (*p* = 0.08) (Figure 1A,C). Moreover, the total activity count was comparable between the Bmal1+/+ control and Bmal1+/+ with an RH (*p* = 0.4). However, Bmal1−/− mice with an RH showed a significant decrease in total activity as compared with Bmal1−/− control mice (*p* = 0.02). In both LL and LD, there were no significant differences in spontaneous locomotor activity between males and females of both genotypes (*p* > 0.05).

### 2.2. Expression of mPER1 and p-ERK in SCN Is Affected by RH

**Figure 2:**
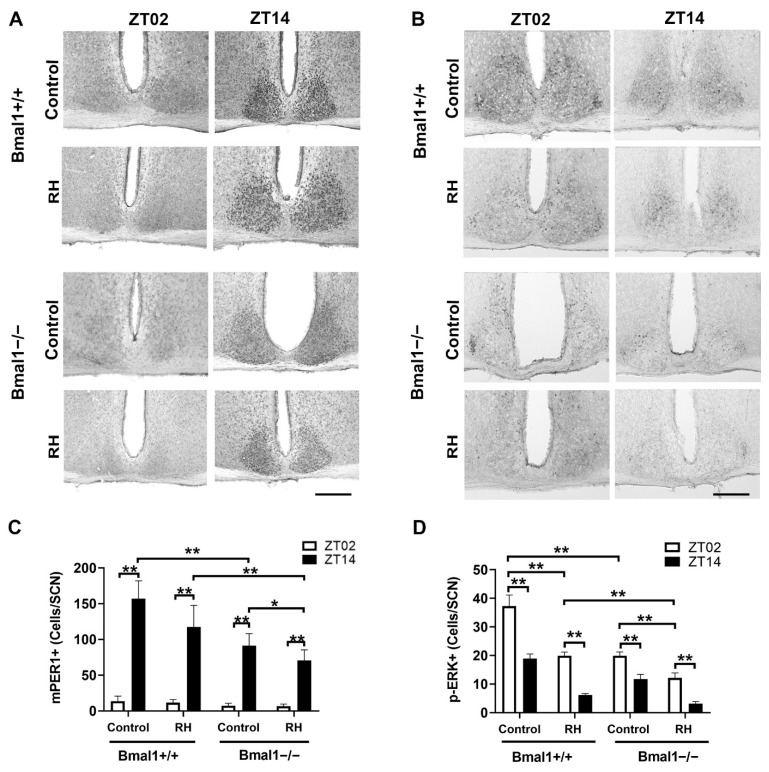
(**A**) Representative mPER1-immunoreactive (+) cells in the SCN of Bmal1+/+ and Bmal1−/− mice perfused at ZT02 or ZT14 without (control) and with RH. Scale bar = 100 µm. (**B**) Representative p-ERK-immunoreactive (+) cells in the SCN of Bmal1+/+ and Bmal1−/− mice without (control) or with RH, perfused at ZT02 or ZT14. Scale bar = 100 µm. (**C**) Quantification of mPER1+ cells in the SCN of the different subgroups. (**D**) Quantification of p-ERK+ cells in the SCN. ZT02 (white bars), ZT14 (black bars). Values are shown as mean + SEM. * *p* < 0.05, ** *p* < 0.01. Groups were compared using the Mann-Whitney U-test (*n* = 6 per subgroup).

The number of mPER1+ cells in the SCN was higher at ZT14 as compared to ZT02 in all subgroups (*n* = 6–7 mice in each subgroup). There was no significant difference in the number of mPER1+ cells between Bmal1+/+ mice with and without RH at ZT02 or at ZT14 (*n* = 6 mice: 3 female and 3 male mice for each condition at each time point). However, the number of mPER1+ cells at ZT14 was significantly lower in the Bmal1−/− controls (*n* = 7 mice: 4 females and 3 males) as compared to the Bmal1+/+ control mice (*p* = 0.001). The number of mPER1+ cells at ZT14 in the SCN of Bmal1−/− mice was significantly lower in the RH group (*n* = 6 mice: 4 females and 2 males) than in the respective control group (*p* = 0.04) or Bmal1+/+ with an RH group (*p* = 0.02) (Figure 2A,C).

The number of p-ERK+ cells in the SCN was higher at ZT02 as compared to ZT14 in all subgroups (*n* = 6–7 mice in each subgroup). In the Bmal1+/+ control group, the number of p-ERK+ cells was higher than in the Bmal1+/+ RH group at both ZT02 and ZT14 (*n* = 6 mice: 3 female and 3 male mice for each condition at each time point) (*p* = 0.002). In the Bmal1−/− control group (ZT02: *n* = 6 mice: 4 females and 2 males; ZT14: *n* = 7 mice: 4 females and 3 males), the number of p-ERK+ cells was lower than in the Bmal1+/+ control group (*n* = 6 mice: 3 female and 3 male mice at each time point) at both ZT02 (*p* = 0.002) and ZT14 (*p* = 0.001). In the Bmal1−/− RH group (ZT02: *n* = 6 mice: 4 females and 2 males; ZT14: *n* = 6 mice: 3 females and 3 males), the number of p-ERK+ cells was lower as compared to the Bmal1−/− control group at ZT02 (*p* = 0.002) and at ZT14 (*p* = 0.0006), and also compared to the respective Bmal1+/+ groups (*p* < 0.01) (Figure 2B,D).

### 2.3. Plasma Corticosterone Level Is Not Affected by RH

The plasma corticosterone level was higher at ZT14 as compared to ZT02 in all subgroups (*n* = 5 per subgroup) (Table 1). There were no significant differences among the subgroups either at ZT02 (*p* = 0.3) or at ZT14 (*p* = 0.9)

## 3. Discussion

The RH is appreciated by nocturnal laboratory rodents as a dark hiding place during the inactive/light phase, and by animal attendants as it allows visual control of the animals without disturbance [46]. Our study shows that this trade-off works well in wildtype mice with an intact light input into the circadian system and an intact molecular clockwork. However, in Bmal1−/− mice, which have a corrupted light input into the circadian system and a disturbed molecular clockwork, the RH has a detrimental effect on the synchronization of rhythmic spontaneous locomotor activity to the standard laboratory light/dark conditions. Thus, for studies on time-of-day-dependent behavior and brain/body functions in mice with impacted visual and/or clockwork function, the use of the RH should be considered carefully.

As expected for nocturnal animals, Bmal1+/+ mice show higher locomotor activity during the dark phase as compared to the light phase. Similarly, and in agreement with our previous observations [39], locomotor activity was higher during the dark phase in Bmal1-deficient controls despite a compromised light input into the circadian system and a disrupted circadian clock [37,40,41,48]. This rhythm is due to the suppression of activity by light, an effect called negative masking, rather than entrainment, which requires an intact clockwork. The use of an RH did not affect rhythmic spontaneous locomotor activity in Bmal1+/+ mice; thus, the RH is applicable for studies on time-of-day-dependent rhythms in wildtype mice. In contrast, the use of the RH in Bmal1−/− mice had a detrimental effect on masking. This is surprising, as the RH filters out the short-wavelength light that is known to have the largest melanopsin-dependent effects on the circadian system. However, the circadian system also receives cone-based chromatic signals [49]. Especially under low-irradiance conditions, longer-wavelength light (L-opsin activation) has a stronger impact on the entrainment of circadian rhythms than shorter-wavelength light (S-opsin activation), while providing identical melanopsin and rod activation [35]. Importantly, negative masking depends on both melanopsin [50] and the classical photoreceptors [51]. Tissue-specific lesions of Bmal1 on the retina affect visual information processing in both the rod and cone pathways [14,41] and reduce the viability of cones, especially in older mice [14]. Thus, for this study, we used mice at the age of 30 weeks, at which age Bmal1−/− mice show symptoms of premature aging [38]. Thus, we conclude that Bmal1−/− are not able to detect the red light at low irradiance, and will interpret the light conditions in an RH as “darkness”, but will be able to detect the light in the home cage with their remaining photoreceptors. We assume that both Bmal1−/− subgroups awoke at a random time, as the circadian rhythm generator was disrupted and, consequently, they were not able to anticipate the dark phase. However, the Bmal1−/− mice sleeping in an RH are faced with “darkness” upon waking and will leave the RH. Outside the RH, they are faced with either light or darkness. If faced with “light”, they are confused by the contradicting information and might return to the RH. If faced with “dark”, they stay awake until the lights are turned on. In contrast, the Bmal1−/− control mice are faced with “light” upon waking during the light phase, which immediately inhibits activity, or with “dark” upon waking during the dark phase. Thus, the higher activity of the Bmal1−/− control group, particularly during the dark phase, is a result of benefiting more efficiently from the “light” and “dark” information.

In the SCN of Bmal1+/+ mice, mPER1-Ir was high during the early dark phase (ZT14) and low at the early light phase (ZT02), as expected [52,53]. This difference was not affected in Bmal1+/+ RH or Bmal1−/− control mice, consistent with rhythmic behavior. The differences in PER1 expression in Bmal1−/− control mice are consistent with our earlier observations that Bmal1 is dispensable for light-induced mPER1 expression. However, the amplitude of mPER1-Ir at ZT14 in Bmal1−/− control mice was reduced as compared to Bmal1+/+ control mice, presumably because, here, the rhythmic expression is driven by light only, and not additionally by the molecular clockwork. Surprisingly, in Bmal1−/− RH mice, mPER1-Ir was high at ZT14 and low at ZT02. This is unexpected, as Bmal1−/− RH mice did not show rhythmic locomotor activity. This suggests that the light which the mice received during the light phase outside the RH is sufficient to drive rhythmic mPER1 expression in the SCN. To further prove this hypothesis, we analyzed p-ERK in the SCN, which is strongly activated by light and plays an important role in the activation of CRE/CREB-mediated transcription [23,54,55]. Indeed, p-ERK-Ir was higher at ZT02 as compared to ZT14 in all four subgroups. Interestingly, the amplitude of p-ERK-Ir in Bmal1+/+ RH mice at ZT02 was lower as compared to Bmal1+/+ control mice, and similar to that in Bmal1−/− control mice. In Bmal1−/− mice with an RH, the amplitude of p-ERK-Ir at ZT02 was even lower. This suggests that the number of p-ERK+ SCN cells is correlated with the strength of the light input. Moreover, the data indicate that the light that the Bmal1−/− mice receive during the light phase outside the RH is sufficient to induce p-ERK expression in the SCN, independent of rhythmic activity. Moreover, plasma corticosterone levels were higher at ZT14 as compared to ZT02 in all four subgroups. This is consistent with the observations of Sollas et al. [56], showing that both the light/dark cycle and the activity rhythm contribute to rhythmic corticosterone levels, and that the rhythm of clock gene expression in the SCN is aligned with the rhythm of clock expression in the adrenal cortex. In addition, there was no difference in corticosterone levels at ZT02 or ZT14 among all subgroups, indicating that mice without an RH do not suffer from higher stress levels.

Taken together, under standard laboratory light/dark conditions, the application of the RH in Bmal1−/− mice, which have both circadian dysfunction and light-input impairment, affects rhythmic spontaneous locomotor activity and dampens the rhythmic expression of mPER1 and p-ERK in the SCN. On the other hand, mice with and without the RH have similar glucocorticoid levels, suggesting little impact on chronic stress. Thus, for studies using mice with a corrupted circadian system, we recommend not using the RH. Moreover, this study shows that the rhythmic expression of mPER1 and p-ERK in the SCN is less prone to impairment of retinal function and independent of rhythmic activity.

## 4. Materials and Methods

### 4.1. Animals

Adult male (*n* = 22) and female (*n* = 27) Bmal1−/− and wildtype (Bmal1+/+) littermates obtained by breeding of male and female Bmal1+/- (generously provided by Christopher Bradfield [37]) were kept individually in standardized type 2 short cages in temperature- and light-controlled (250–300 lux) cabinets (ScanClime^BASIC^, Scanbur, Denmark, light bulb: Osram 6428 12V 3W SV7, Osram, Germany) and were fed ad libitum. All animal experiments were conducted in accordance with international guidelines for the ethical use of experimental animals.

### 4.2. Behavioral Analysis

The mice were randomly divided into two groups. One group was kept without the RH (control), and one with the RH (Zoonlab GmBH, Castrop-Rauxel, Germany; Figure 1B). All mice were checked daily at different times during the light phase, without disturbing them, to ensure that the RH mice used the RH to sleep in. The mice were kept under a standard 12-h light/12-h dark (LD, lights on at 06:00 am and lights off at 06:00 pm) cycle for 3 weeks.

The light intensity under the RH was 35 lux, as determined using a luxmeter, and the light wavelength was >600 nm, as determined using a Varian Cary^®^ 50 UV-Vis Spectrophotometer. During the dark phase, the light intensity was 0 lux either with or without the RH. This was followed by a period of constant light (LL) for 3 weeks, and a second LD cycle for an additional 3 weeks. LL was used for desynchronization of SCN clock cells [57,58] to better evaluate the potential of the ambient light conditions to re-synchronize the respective rhythms. Spontaneous locomotor activity was continuously recorded using on-cage infrared detectors (Mouse-E-Motion, Hamburg, Germany) and was analyzed using Clocklab software (Actimetrics, Wilmette, IL, USA).

### 4.3. Tissue Processing

After the second LD, each group was divided into two subgroups that were sacrificed 2 h after lights on (ZT02) or 2 h after lights off (ZT14) using an overdose of ketamin/xylazine (100 and 10 mg/kg, respectively). The mice were perfused transcardially with 0.09% NaCl and then with 4% formaldehyde using a Ministar Peristaltic Pump (World Precision Instruments, Sarasota, FL, USA); then, brains were removed from the skull, underwent post-fixation for an additional 24 h, and were cryoprotected in 30% sucrose until sinking. The brains were sectioned through the whole rostro-caudal extent of the SCN using a cryostat (Leica CM, Wetzlar, Germany), to obtain a series of 30-µm-thick free-floating coronal sections. The sections were kept at −20 °C until further processing.

### 4.4. Immunohistochemistry

The sections were rinsed in PBS, then incubated in 5% normal goat serum in PBS-T 0.2% for one hour at RT to block the nonspecific binding of secondary antibody, followed by incubation with markers for rhythmic SCN neuronal activity rabbit monoclonal anti phosphorylated ERK1/2 (p-ERK) (1:1000, #4695, Cell Signaling Technology, Danvers, MA, USA) and rabbit polyclonal mPER1 (1:3000, #P3342-11, US Biologicals, USA), as described previously [59]. The sections were incubated with the VECTASTAIN Elite ABC HRP Kit (Vectorlabs, Burlingame, CA, USA) for one hour at RT, then rinsed and incubated with 0.05% 3, 3′-diaminobenzidine (Sigma-Aldrich, St. Louis, MO, USA) for 5 min. Finally, the sections were cover-slipped using entelan.

### 4.5. Image Analysis

All images were acquired with bright field microscopy (BZ-9000E, Keyence, Japan) using identical settings. All images were equally subjected to image processing, including haze reduction for contrast enhancement. The number of p-ERK- and mPER1-immunoreactive (+) cells within the unilateral SCN was counted in a blinded manner using Image J software. For each mouse, the average number of p-ERK+ or mPER1+ cells per SCN was calculated from three sections of the respective series.

### 4.6. Plasma Corticosterone Analysis

Blood was collected from the right atrium in EDTA tubes and centrifuged. Plasma corticosterone levels were analyzed using the enzyme-linked immunosorbent assay (ab108821, Abcam), according to the manufacturer’s instructions.

### 4.7. Statistics

Statistical analysis was performed using Graph Pad Prism software. The Mann-Whitney U-test was used to determine differences between two groups, and the Kruskal-Wallis test to compare multiple groups. A *p* value < 0.05 was considered statistically significant. Values were presented as the mean ± SEM.

## Figures and Tables

**Table 1:** Plasma corticosterone level (ng/mL) during the early inactive phase (ZT02) and early active phase (ZT14). The *p* value indicates differences between ZT02 and ZT14 in each subgroup.

	Bmal1+/+ Control	Bmal1+/+ RH	Bmal1−/− Control	Bmal1−/− RH
ZT02	2.8 ± 0.9	2.7 ± 0.5	1.9 ± 0.4	1.6 ± 0.2
ZT14	7.1 ± 0.1	6.9 ± 0.9	6.6 ± 1.4	7.6 ± 0.7
*p*-value	0.03	0.008	0.02	0.008

## Supplementary Materials

The following are available online at https://www.mdpi.com/1422-0067/22/5/2288/s1, Figure S1: (A) Quantitative analysis of total spontaneous locomotor activity counts during constant light (LL). **: *p* < 0.01, ***: *p* < 0.001. The power of the 24 h phase was analyzed by fast Fourrier transformation (FFT) in (B) Bmal1+/+ and (C) Bmal1−/− without (black) and with (red) RH during the first 10 days after transition to LL.
