# Supplementary material for: Does a Red House Affect Rhythms in Mice with a Corrupted Circadian System?
Source: Int J Mol Sci. 2021 Feb 25;22(5):2288. doi: 10.3390/ijms22052288 (PMC7956239; doi:10.3390/ijms22052288)
Supplement: Supplementary file 1 [file ijms-22-02288-s001.pdf]

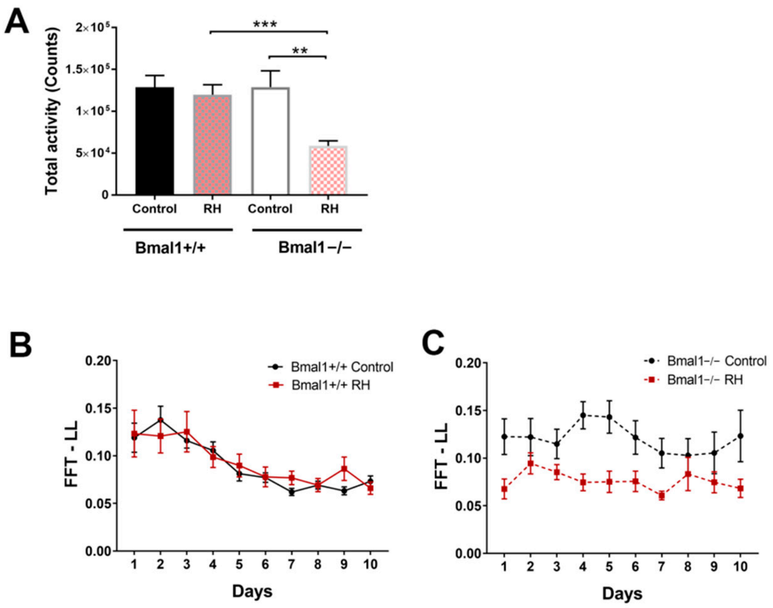

Suppl. Fig1. (A) Quantitative analysis of total spontaneous locomotor activity counts during constant light (LL). **: $p < 0.01$, ***: $p < 0.001$. The power of the 24 h phase was analyzed by fast Fourrier transformation (FFT) in (B) Bmal1+/+ and (C) Bmal1-/- without (black) and with (red) RH during the first 10 days after transition to LL. The area under the curve in Bmal1-/- mice with RH is significantly smaller than in the Bmal1-/- control group ($p < 0.001$).
